# *In vitro* and *in vivo* toxicological evaluation of carbon quantum dots originating from *Spinacia oleracea*

**DOI:** 10.1016/j.heliyon.2023.e13422

**Published:** 2023-02-02

**Authors:** Cuicui Fu, Xiaoyun Qin, Jin Zhang, Ting Zhang, Yeqing Song, Jiaqi Yang, Gang Wu, Dan Luo, Nan Jiang, Floris J. Bikker

**Affiliations:** a Department of Oral Biochemistry, Academic Centre for Dentistry Amsterdam (ACTA), University of Amsterdam (UvA) and Vrije Universiteit Amsterdam (VU), Amsterdam 1081LA, the Netherlands; b School of Material and Chemical Engineering, Zhengzhou University of Light Industry, Zhengzhou 450002, China; c Laboratory of Biomimetic Nanomaterials, Department of Orthodontics, Peking University School and Hospital of Stomatology, National Engineering Laboratory for Digital and Material Technology of Stomatology, Beijing Key Laboratory of Digital Stomatology, Beijing 100081, China; d Central Laboratory, Peking University School and Hospital of Stomatology, #22 Zhongguancun, South Avenue, Haidian District, Beijing 100081, China; e Shanxi Medical University School and Hospital of Stomatology& Shanxi Province Key, Laboratory of Oral Diseases Prevention and New Materials, Shanxi 030605, China; f Department of Oral and Maxillofacial Surgery/Pathology, Amsterdam UMC and Academic, Center for Dentistry Amsterdam (ACTA), Amsterdam Movement Science, Vrije Universiteit Amsterdam, Amsterdam 1081LA, the Netherlands; g Department of Oral Cell Biology, Academic Center for Dentistry Amsterdam (ACTA), University of Amsterdam and Vrije Universiteit Amsterdam, Amsterdam 1081LA, the Netherlands; h CAS Center for Excellence in Nanoscience, Beijing Key Laboratory of Micro-nano Energy and Sensor, Beijing Institute of Nanoenergy and Nanosystems, Chinese Academy of Sciences, Beijing 101400, China; i School of Nanoscience and Technology, University of Chinese Academy of Sciences, Beijing 100049, China

**Keywords:** Food safety, *Spinacia oleracea*, Carbon quantum dots, Toxicity, Fluorescence

## Abstract

Food-derived carbon quantum dots (CQDs) can relatively easily be synthesized and chemically manipulated for a broad spectrum of biomedical applications. However, their toxicity may hinder their actual use. Here, *Spinacia oleracea*-derived CQDs *i.e*., CQD-1 and CQD-2, were synthesized by means of different shredding methods and followed by a microwave-assisted hydrothermal approach. Subsequently, these CQDs were analyzed *in vitro* and in an *in vivo* mice model to test their biocompatibility and potential use as bioimaging agents and for activation of osteogenic differentiation.

When comparing CQD-1 and CQD-2, it was found that CQD-1 exhibited 7.6 times higher photoluminescent (PL) emission intensity around 411 nm compared to CQD-2. Besides, it was found that the size distribution of CQD-1 was 2.05 ± 0.08 nm, compared with 2.14 ± 0.04 nm for CQD-2. Upon exposure to human bone marrow-derived mesenchymal stem cells (hBMSCs) *in vitro*, CQD-1 was endocytosed into the cytoplasm and significantly increased the differentiation of hBMSCs up to 10 μg mL^−1^ after 7 and 14 days. Apparently, the presence of relatively low doses of CQD-1 showed virtually no toxic or histological effects in the major organs *in vivo*. In contrast, high doses of CQD-1 (1 mg mL^-1^) caused cell death *in vitro* ranging from 35% on day 1 to 80% on day 3 post-exposure, and activated the apoptotic machinery and increased lymphocyte aggregates in the liver tissue. In conclusion, *S. oleracea*-derived CQDs have the potential for biomedical applications in bioimaging and activation of stem cells osteogenic differentiation. Therefore, it is postulated that CQD-1 from *S. oleracea* remains potential prospective material at appropriate doses and specifications.

## Highlights

• *QY* value of *S. oleracea-*CQD-1 was 7.3%, compared with 1.0% for *S. oleracea-*CQD-2.
• CQD-1 were taken into cytoplasm and their LC50 is 400.6 μg mL^−1^ at 3 days.
• CQD-1 activated osteogenesis of stem cells at the concentration of 1 μg mL^−1^.
• CQD-1 induced no histological changes at low doses *in vivo.*
• *S. oleracea-*CQDs are relatively safe and applicable for biomedical applications.

## Graphical abstract

**Figure:**
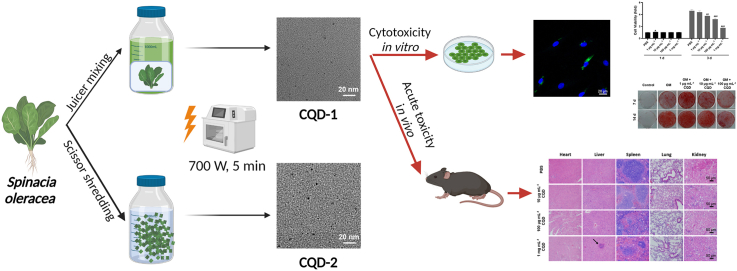

## 1 Introduction

Recently, the health risks of baked food-related substances have attracted increasing attention. Thermal processing such as baking, frying, and roasting not only improves food digestion and accelerates absorption, but also improves flavors and kills bacteria [1]. During thermal processing, foods are given their typical color and flavor by the Maillard reaction, which is a chemical reaction between compounds such as amino acids and carbohydrate moieties. As a result of this reaction, toxicants that are potentially mutagenic and carcinogenic, are formed [2]. For example, heterocyclic aromatic amines (HAAs), which are found in grilled meat and fish, are known to play a crucial role in the etiology of cancer [3]. Next, acrylamide, known to have neurotoxic, hepatotoxic, and genotoxic effects, is found after high-temperature processes such as cooking, frying, toasting, roasting, or baking in carbohydrate-rich foods in particular [4]. Therefore, it is very important to explore and evaluate the systematic toxicity of products formed during thermal processes.

Also, carbon quantum dots (CQDs) emerge in thermally processed food such as roast duck breasts [5], beer [6], and baked lamb [7]. Due to their small dimensions (< 10 nm) and relatively large surface areas, CQDs are easily ingested and absorbed by the human body evoking, to some extent, (cyto)toxic effects [8]. For example, CQDs from roasted chicken have shown dose-dependent cytotoxic effects by causing lysosomal damage and mitochondrial dysfunction, which led to apoptosis and necrosis [9]. CQDs found in processed Atlantic salmon accumulated in the brain, kidney, intestines, and liver of mice after oral feeding [10]. Interestingly, CQDs are widely researched and applied for a broad spectrum of biomedical purposes including labeling, bioimaging, detection of heavy metals, anti-tumor, and therapeutics [11,12] due to their exclusive characteristics such as tunable fluorescence, high biocompatibility, and physiochemical stability [13].

CQDs can be synthesized not only from food substances [14,15] but also from chemical precursors [[16], [17], [18]]. So far, various synthesis approaches have been developed for the preparation of CQDs, *i.e.*, top-down methods such as arc discharge, laser ablation, electrochemical oxidation, and chemical oxidation; and bottom-up methods such as microwave synthesis, thermal decomposition, and hydrothermal treatment [19], which are the usual methods of generating CQDs from food substances. These methods have various advantages and disadvantages. Microwave-assisted method provides simultaneous, homogeneous heating, which leads to uniform size distribution of CQDs [20,21]. While thermal decomposition offers advantages of a solvent-free approach, and wide precursor tolerance, it leads to a non-uniform distribution of CQDs [22]. Similarly, while hydrothermal carbonization is an environmentally friendly route [23], the yield of CQDs is generally low [24].

To date, evaluations of the toxicity of CQDs derived from food via thermal processes have covered only a limited range of foods. *S. oleracea* is very versatile since it is commonly used as a cooked vegetable, or as a component of many other cooked meat and vegetable dishes [25], yet the toxicity of *S. oleracea*-derived CQDs has not been analyzed so far. Here, using commonly known cooking procedures, *i.e.,* manual as well as automated crushing followed by microwave-assisted heating CQDs from *S. oleracea* were generated and their potential (cyto)toxicity was evaluated i*n vitro* and *in vivo*. After initial structural and morphological characterization of CQD-1 and CQD-2, CQD-1 was chosen for further evaluation. The cytotoxicity of CQD-1 *in vitro* was evaluated via CCK-8, cell apoptosis. Besides, the application of CQD-1 in stem cells osteogenic differentiation was investigated. Furthermore, *in vivo* toxicity was evaluated in mice via an acute toxicity model.

## 2 Materials and methods

### 2.1 Preparation of CQDs from *Spinacia oleracea*

CQDs were prepared from *S. oleracea* in two ways denoted CQD-1 and CQD-2. For CQD-1, 100 g *S. oleracea* and 1 L water were added to a juicer (Joyoung, Y88, Hangzhou, China) and mixed for 5 min at room temperature (RT), and the slurries subsequently underwent microwave irradiation (Media, Foshan, China) of 700 W for 5 min. For CQD-2, 100 g *S. oleracea* were shredded by a scissor to particles with an approximate size of 2 cm^2^, mixed with 1 L water and transferred to microwave for irradiation as well. Then, both slurries were collected and centrifuged at 14,000 g for 10 min using TG16-W centrifuge (Cenlee, Changsha, China) and the supernatant was collected for further treatment. Both fluids, contained with CQDs, were dialyzed against dH_2_O through a dialysis membrane (molecular weight cutoff, 1000 Da, ACMEC biochemical, Shanghai, China) for 2 d and refreshed dH_2_O every 12 h. The liquid was dried overnight (ON) with a NAI Freeze-drier, T3-50 (Xinyu, Shanghai, China), and the obtained solids were weighed to calculate the yield of CQDs. Finally, the CQDs were dispersed in dH_2_O with a concentration of 2.0 mg mL^−1^ for further characterization.

### 2.2 Characterization of CQDs

Fluorescent emission spectra were recorded on a RF-5301PC spectrofluorometer (Shimadzu, Tokyo, Japan). Fourier transform infrared (FT-IR) spectra were performed on an IFS 66V/S (Bruker, Billerica, USA) IR spectrometer in the range of 400–4000 cm^−1^. X-ray photoelectron spectroscopy (XPS) analysis was measured on an ESCALAB MK II X-ray photoelectron spectrometer using Mg^2+^ as the exciting source. UV–vis spectra were obtained on a UV-1800 spectrophotometer (Shimadzu). Transmission electron microscopy (TEM) measurements were made on a HITACHI H-8100 electron microscopy (Hitachi, Tokyo, Japan) with an accelerating voltage of 200 kV. The sample for TEM characterization was prepared by placing a drop of colloidal solution on carbon-coated copper grid and dried at RT. Quantum yield (*QY*) was measured according to the following equation, $QY=QY_{std}\frac{m}{m_{std}}\frac{\eta^{2}}{\eta_{std}^{2}}$ where *m* and *mstd* are the slopes of samples and standards. *η* and *ηstd* are the refractive index of solvents. Quinine sulfate in 0.1 M H_2_SO_4_ (*QYstd* = 0.54 at 360 nm) was chosen as standard. To minimize re-absorption effects, the absorbance in the 10 mm quart cuvette was kept under 0.1 at the excitation wavelength of 360 nm.

### 2.3 Cell culture

Human Bone Marrow Mesenchymal Stem Cells (hBMSCs) were obtained from the Central Laboratory, School and Hospital of Stomatology, Peking University. hBMSCs were cultured in alpha-minimum essential medium (α-MEM; Invitrogen, Carlsbad, CA, USA) supplemented with 10% fetal bovine serum (FBS; Invitrogen, Carlsbad, CA, USA) and 1% penicillin/streptomycin (Sigma, St. Louis, MO, USA). Cells were cultured in humidified oxygen-controlled 37 °C incubator with 5% CO_2_. All the subsequent experiments were performed using the CQD-1.

### 2.4 Cytotoxicity assessment

CCK-8 (Cell counting kit-8, SAB biotech. College Park, MD, USA) assays were used to evaluate the *in vitro* cytotoxicity of CQD-1. Briefly, hBMSCs (1 × 10^4^ well^−1^) were seeded on a 96-well plate (Thermo Fisher, Massachusetts, USA) and treated with PBS as control, and 1, 10, 100, and 1 mg mL^−1^ CQD-1. After 1 d and 3 d incubation, cell proliferation was analyzed with a CCK-8 reagent following the manufacturer's instructions. Optical density (OD) was recorded at 450 nm using ELx808 (Lonza, Basel, Switzerland). The cells' relative survival rate (mean ± SD) was calculated using the equation: survival rate = A450 treated with CQDs group/A450 PBS group on day 1.

### 2.5 Osteogenic differentiation experiments

To test the effect of CQD-1 on hBMSCs’ differentiation, hBMSCs were cultured in osteogenic medium (OM) containing 10 mM β-glycerophosphate, 100 μM l-ascorbic acid 2-phosphate, and 10 nM dexamethasone (Sigma). Cells were cultured in the presence of PBS (Control group), OM (OM group), 1 μg mL^−1^ CQD (OM + 1 μg mL^−1^ CQD group), 10 μg mL^−1^ CQD (OM + 10 μg mL^−1^ CQD group), and 100 μg mL^−1^ CQD (OM + 100 μg mL^−1^ CQD group) for 7 d and 14 d. To observe mineralized nodules, hBMSCs were stained with 1% Alizarin Red S (Sigma) for 15 min at RT to detect mineral nodules after being fixed with 4% PFA for 1 h at 4 °C, after 7 d and 14 d incubation with conditioned medium. The stained cells were observed under the TI-S inverted microscope (Nikon, Tokyo, Japan) at 10× magnification. Experiments were performed in triplicate and repeated at least 3 times.

### 2.6 Flow cytometric analysis of the cell apoptosis

Cellular apoptosis analysis was performed with the hBMSCs seeded at 1 × 10^5^ cells mL^−1^ in a 6-well plate (Corning, New York, USA). When cells reached 70% confluence, they were incubated with CQD-1 at a concentration of 10 μg mL^−1^, 100 μg mL^−1^, 1 mg mL^−1^, or PBS for 1 d and 3 d, respectively. To induce apoptosis, 5 μM camptothecin (Sigma) was added to the cells for 3 h as a positive control group. The hBMSCs were harvested by trypsinization and centrifugation at 1200 rpm for 5 min, washing thoroughly with 1 mL cold PBS. Cells were stained following the FITC Annexin V Staining Protocol (BD Bioscience, USA) to measure apoptosis by flow cytometry. Briefly, cells were incubated with 5 μl of FITC Annexin V and 5 μl PI for 15 min at RT in the dark. Apoptosis was analyzed by flow cytometry within 1 h using FACSAria II (BD, New Jersey, USA) as described earlier [26].

### 2.7 Endocytosis of CQDs

The hBMSCs were transferred onto 12-well plates (Corning) at a density of 1 × 10^5^ cells well^−1^ and incubated at 37 °C, in 5% CO_2_ humidified atmosphere. When the confluence of cells reached 80%, PBS and CQDs were respectively added into the growth media at the final concentration of 10 μg mL^−1^ separately, for 12 h at 37 °C. Subsequently, the cells were washed with pre-warmed PBS, and then the cellular fluorescence was observed by a Zeiss laser scanning confocal microscope LSM 710 (Zeiss, Oberkochen, Germany).

### 2.8 Acute toxicity model

Six-week-old male C57BL/6 mice were purchased from Peking University Health Science Center (Peking, China). 20 mice were randomly divided into 4 groups: 1) control group receiving PBS, 2) group receiving 10 μg mL^−1^ CQDs, 3) group receiving 100 μg mL^−1^ CQDs, and 4) group receiving 1 mg mL^−1^ CQDs. Each mouse was orally gavaged with 200 μl once every two days by PBS, 10 μg mL^−1^ CQDs, 100 μg mL^−1^ CQDs, and 1 mg mL^−1^ CQDs, respectively. The animal study was approved by the Peking University Institutional Animal Care and Use Committee (Approval number: LA2021-036). After 2 weeks of oral gavaging, C57BL/6 mice were used to investigate the acute toxicity of CQDs *in vivo.*

### 2.9 Evaluation of toxicity *in vivo*

All mice were sacrificed by over-anesthesia, and the main organs: heart, liver, spleen, lungs, and kidneys were obtained and immediately fixed with 4% paraformaldehyde. Organ specimens were processed following typical methods, including infiltration with paraffin, clearing with xylen, and dehydration with ethanol followed by exposure to a graded series of ethanol (70–100%) as described [27]. Paraffin-embedded blocks were sectioned to 5-μm thickness by Microtome (Leica, Wetzlar, Germany). Randomly selected sections from one individual organ were collected and used for hematoxylin and eosin (H&E) staining for inflammation analysis, essentially as described elsewhere [28]. The stained slides were observed under a Zeiss light microscope.

### 2.10 Statistic analysis

All data are presented as mean ± standard deviation (SD). Each experiment was repeated three times. The data were analyzed by one-way ANOVA by GraphPad Prism 8.0 (GraphPad Software Inc., La Jolla, CA, USA). LC50 values were the best-fitted value of a nonlinear regression using the logistic equation with the GraphPad Prism 8.0. A p value of < 0.05 was considered statistically significant.

## 3 Results and discussion

### 3.1 Structural characterization of CQD-1 and CQD-2

**Fig. 1:**
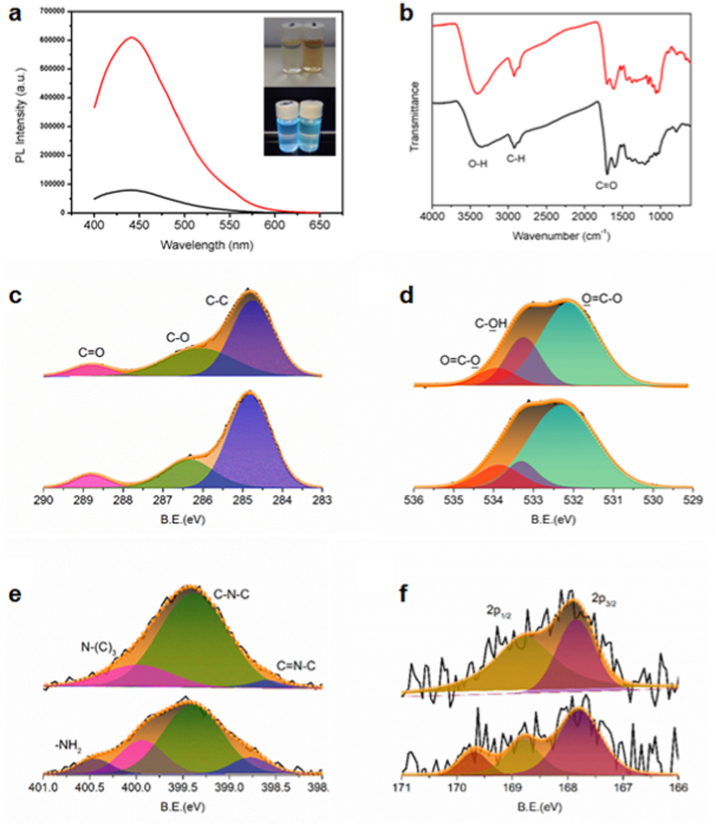
Structural characterization of CQDs. (a) The photoluminescent (PL) spectra of CQD-1 (red curve) and CQD-2 (black curve) at 10 μg mL^−1^. The inset exhibits the optical photo of CQD-1 (left) and CQD-2 (right) at the concentration of 1 mg mL^−1^ under irradiation of natural light (top) and UV 365 nm light (bottom). (b) The FTIR spectra of CQD-1 (red curve) and CQD-2 (black curve). High resolution XPS spectra of (c) C 1s, (d) O 1s, (e) N 1s, and (f) S 2p for CQD-1 (top) and CQD-2 (bottom), respectively.

We generated two novel types of CQDs extracted from *S. oleracea* denoted CQD-1 and CQD-2. The microwave-assisted carbonization produced yellow solutions with blue fluorescent emission under UV irradiation (Fig. 1a). The FT-IR spectra (Fig. 1b) indicated the similar surface functional groups for the two CQDs, for the presence of a broad peak around 3300 cm^-1^ indicating the hydroxyl groups, 2920 cm^-1^ C–H groups, and 1700 cm^-1^ carbonyl groups. The abundant vitamins and carbohydrates in spinach potentially ensure the rich surface oxidation groups to endow the CQDs with good water solubility, while the green carbon source from biomass and absolute nontoxic synthesis gives the potential biocompatibility for subsequent biomedical applications. CQD-1 produced from automated shredded spinach exhibited 7.6 times higher photoluminescent (PL) emission intensity around 411 nm compared with CQD-2 originating from manually shredded spinach (Fig. 1). The bright emission from CQDs suggested *e.g.,* a bioimaging application as a fluorescent tracer or visual drug microcarrier *in vivo*. In accord with previous reported CQDs, CQD-1 behaved an excitation-dependent PL emission spectrum ranging from 420 to 500 nm at excitation from 330 to 420 nm, which may refer to different emissive traps caused by size distribution and inconsistent surface chemistry of CQDs [29].

The two kinds of CQDs possessed a comparable element partitioning ratio, as shown in Table S1. However, CQD-1 exhibited a slightly higher heteroatomic percentage (21.65 at% for CQD-1 and 20.09 at% for CQD-2), which properly derived from more oxidation state and higher binding energy compared with CQD-2, as shown in Fig. 1c–f and Table S2. The dominant deconvolution peak from the C1s spectrum located at 284.77 eV for CQD-1 and 284.82 eV for CQD-2 were assigned to graphitic sp^2^ C–C type, whereas less area percentage of C–C split peak indicated the more oxidized form existing in the thoroughly crushed carbon precursor (Fig. 1c). Fig. 1d exhibited the O1s spectra of CQDs, while the deconvolution peak can be assigned to O=C–O (534.13 eV for CQD-1, 533.81 eV for CQD-2), C–OH (533.36 eV for CQD-1, 533.21 eV for CQD-2), O=C–O (532.21 eV for CQD-1, 532.09 eV for CQD-2), respectively. Similar situations were found in the N 1s spectrum that much lower content of C=N–C moiety in CQD-1, suggesting more oxidation state of N element produced in the carbonation of spinach juice (Fig. 1e). Tiny S element existed in CQDs, as shown in Fig. 1f. The higher content of functional oxidation groups in CQD-1 contributed to higher *QY* value of as calculated as 7.3% as compared with 1.0% for CQD-2.

### 3.2 Morphological characterization of CQD-1 and CQD-2

**Fig. 2:**
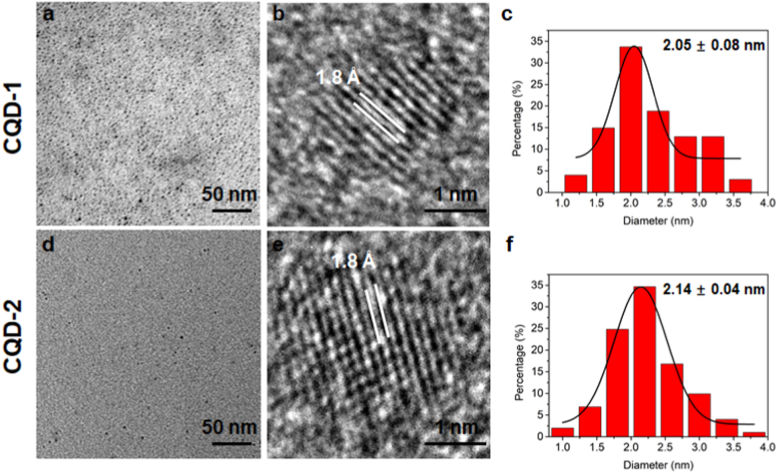
Morphological characterization of CQDs. Low-, and high-resolution TEM images and particle size distributions of (a–c) CQD-1 and (d–f) CQD-2. The synthesized CQD-1 exhibited a smaller size distribution of 2.05 ± 0.08 nm, compared with 2.14 ± 0.04 nm for CQD-2.

TEM characterizations were performed on the typical nanodot morphology of CQD-1 and CQD-2 as shown in Fig. 2a and d, respectively. The high-resolution TEM images (Fig. 2b, e) demonstrated that the two kinds of CQDs possessed clear lattice fringe corresponding to the graphite facet [30], indicating the microwave irradiation created carbonization of vitamins and carbohydrates to produce nanosized graphite core [31,32]. The synthesized CQD-1 exhibited a smaller size distribution of 2.05 ± 0.08 nm (Fig. 2c), compared with 2.14 ± 0.04 nm for CQD-2 (Fig. 2f). Incorporating the above analysis, there were no significant differences in the sizes and surface areas between automatically shredded spinach (CQD-1) and manually shredded spinach (CQD-2), however, CQD-1 exhibited higher PL emission intensity and high *QY* value. Therefore, in order to investigate toxicity and further applications of CQDs from shredded spinach, CQD-1 was therefore selected for subsequent *in vitro* and *in vivo* physiological toxicological analysis.

### 3.3 Endocytosis of CQD-1 and cell labeling

**Fig. 3:**
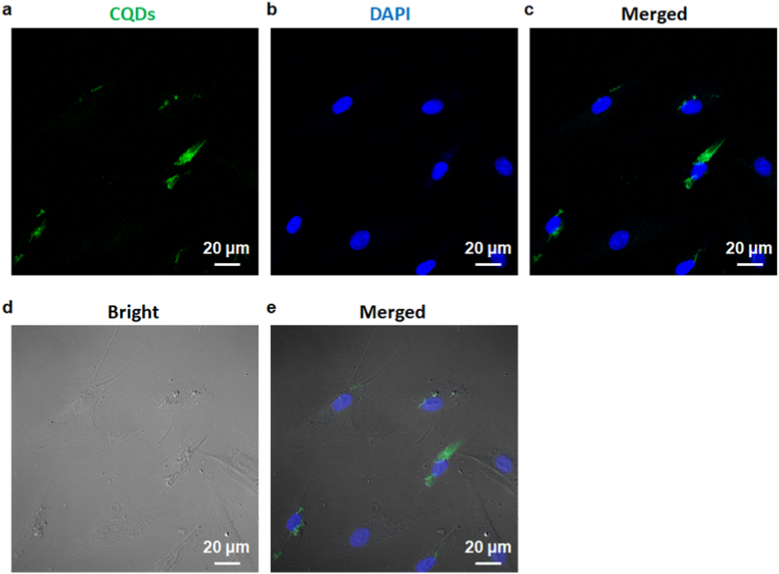
Endocytosis of CQD-1. Confocal images of hBMSCs co-incubated with CQDs for 24 h. Green represents CQDs (a), DAPI (blue) represents the nuclei (b), images merged with CQDs and DAPI (c), bright images (d), and bright images merged with CQDs and DAPI (e). hBMSCs treated with CQD-1 showed green fluorescence using a 488 nm laser indicating CQD-1 had been taken by the cells.

In order to further explore the potential biomedical applications of CQD-1 from *S. oleracea*, it is critical to determine their cellular uptake activity. hBMSCs were incubated with 10 μg mL^−1^ CQDs for 24 h, and their endocytosis was measured by confocal microscopy. As shown in Fig. 3a–c, cells treated with the CQD-1 solution showed green fluorescence when stimulated with a 488 nm laser, and the cell nucleus was stained with DAPI to exhibit bright blue fluorescence under 430 nm excitation. The cell morphology was clearly recognized in the bright field image shown in Fig. 3d. The merged image shown in Fig. 3e suggested that 100% of cells were positive for green fluorescence, indicating CQD-1 had been taken by the cells. The result indicated that *S. oleracea-*CQDs may be a useful probe for intracellular detection. Sharma *et al.* synthesized CQDs from *Calotropis gigantea* by microwave-assisted hydrothermal method and used them as a fluorescent staining approach for optical and bio-imaging of bacteria, fungi, and plant cells [33]. According to the previous report, we also found that *S. oleracea-*CQDs by microwave-assisted hydrothermal method exhibited the potential labeling for *in vivo* tracking applications. Using the report of Liu *et al.* on labeling different cell lines by CQDs, *S. oleracea-*CQDs exert potent promise for a variety of *in vitro* labeling and *in vivo* tracking [34]. However, bioimaging sensitivity or selectivity must be enhanced for the further exploration and application of *S. oleracea-*CQDs to play an essential role in the fluorescence nature.

### 3.4 Effects of CQDs on cell viability and cell apoptosis

Biocompatibility and viability of CQDs are crucial factors that must be considered when investigating nanoparticles for biomedical applications [35,36]. In this light, mesenchymal stem cells (MSCs) have been widely used to test the biocompatibility and bioavailability of CQDs *in vitro* [8,[37], [38], [39]]. MSCs, especially BMSCs, have drawn much attention from basic and translational investigators in the field of regenerative medicine, mainly due to their multipotent differentiation capacity, and immunomodulatory and anti-inflammatory effects [40,41]. Several clinical trials have underscored BMSCs’ effectiveness in treating different illnesses, including liver regeneration, acute myocardial infarction, bone and cartilage, and autoimmune diseases [42,43]. Besides, researchers evaluated the cytotoxic effects of CQDs on hBMSCs [37] and rBMSC [39] *in vitro* and *in vivo*.

#### 3.4.1 Cell viability

**Fig. 4:**
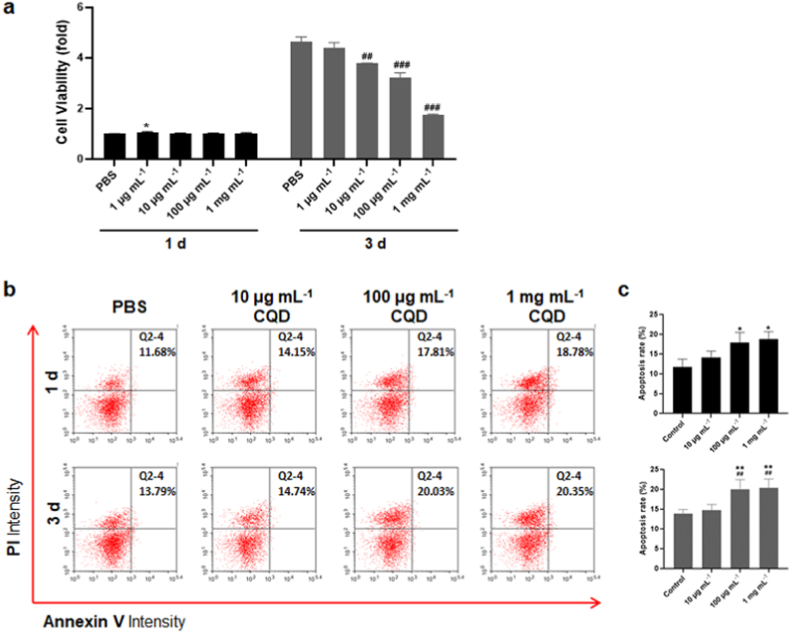
Effects of CQD-1 on cell viability and cell apoptosis. (a) Cytotoxicity evaluations of CQDs by CCK-8 assay on day 1 and day 3. After incubating cells with CQD-1 for 1 day, it was found that the cells proliferation rate virtually remained unchanged when the concentration of CQDs changed from 0 to as high as 1 mg mL^−1^. After extending the observation time to 3 days, cell viability was suppressed after 3 days of incubation with 10 μg mL^−1^, 100 μg mL^−1^, and 1 mg mL^−1^ CQDs. *: P < 0.05 versus PBS group on day 1; ##: P < 0.01; ###: P < 0.001 versus PBS group on day 3. (b) Apoptosis of hBMSCs co-cultured with CQDs on day 1 and day 3. (c) Quantitative analysis of apoptotic hBMSCs on day 1 and day 3, respectively. *: P < 0.05 versus Control 1 d; **: P < 0.01 versus Control 3 d; ##: P < 0.01, versus 10 μg mL^−1^ group on day 3.

To study the cytotoxicity effects of the CQD-1, CCK-8 assay was used to evaluate cell viability on hBMSCs for 1 day and 3 days (Fig. 4a). After incubating cells with CQD-1 for 1 day, it was found that the cells proliferation rate remained virtually unchanged when the concentration of CQDs changed from 0 to as high as 1 mg mL^−1^, only with 1 μg mL^−1^ CQDs, the cell viability increased to 1.07 times higher compared to the control (Fig. 4a). This indicated that CQDs had no cytotoxicity before the concentration reached 1 mg mL^−1^ at 1 day. After extending the observation time to 3 days, Fig. 4a indicated that cell viability decreased after 3 day incubation with 10 μg mL^−1^, 100 μg mL^−1^, and 1 mg mL^−1^ CQDs. The results demonstrated that cell viability had no significant difference between PBS group and the 1 μg mL^−1^ group, while the cell viability in the 100 μg mL^−1^ group decreased to 3.2 times compared with the control group (4.6 times). However, after incubating with 1 mg mL^−1^ CQDs for 3 days, the viability of MSCs reduced to 35% compared with PBS.

Overall, the results show that *S. oleracea-*CQDs exhibited very low cytotoxicity towards hBMSCs with a LC50 of 400.6 μg mL^−1^ for 3 days. Interestingly, CQDs generated from ascorbic acid were found to have mild cytotoxicity after exposure to 80 μg mL^−1^ CQDs [38], and cells exposed to the higher concentration of CQDs (276 μg mL^−1^) show a significant decrease in proliferation after 1 day [44], which suggests that CQDs synthesized from *S. oleracea* have milder negative side-effects on cell proliferation. Interestingly, comparable data were found in a study on CQDs from cabbage: the purified CQDs exhibited low cytotoxicity at relative high concentration (500 μg mL^−1^) during a cell viability experiment against HaCaT cell, an immortalized non-tumerogenic human keratinocyte cell [45]. Based on the current observations, we feel it tempting to postulate that CQD-1 derived from *S. oleracea* potentially is safe for human applications.

#### 3.4.2 Cell apoptosis

Next, we assessed the rate of apoptosis using Annexin V-FITC flow cytometry following 1 d, and 3 d of PBS and different concentrations (10 μg mL^−1^, 100 μg mL^−1^ and 1 mg mL^−1^) CQDs treatment. The results indicated that the apoptotic rate of hBMSCs had no obvious change between the PBS group and 10 μg mL^−1^ group on both 1 d and 3 d (Fig. 4b). After treatment with 100 μg mL^−1^ and 1 mg mL^−1^ of CQDs, there was an increase of 6.13% and 7.10% in the proportion of apoptotic cells on day 1, 6.24%, and 6.56% on day 3, respectively (Fig. 4c). In addition, the proportion of apoptotic cells in the cells treated with CQDs increased from 14.74% to 20.35% with the increase of the CQD concentration from 10 μg mL^−1^ to 1 mg mL^−1^ on day 3 (Fig. 4c). Accordingly, the proportion of apoptotic showed that the 1 mg mL^−1^ CQDs are more toxic than the 10 μg mL^−1^ CQDs. Others found that after exposure to 1 mg mL^−1^ CQDs from roast salmon for 12 h, the percentage of apoptotic cells was 19.65% [46]. Besides, it has been reported that CQDs from graphite plates induced apoptosis of 143B cells to 50% after 3 days and increased apoptotic protein expression [44]. In addition to this, CQDs prepared from ginger induced 43.1% HepG2 cell apoptosis [47]. In line with the previous report, our results showed that CQDs from *S. oleracea* induced low hBMSCs apoptosis after 1 day and 3 days of incubation, which means CQDs exert low cytotoxicity for promising application when you increase the incubation time to 3 days.

### 3.5 Effects of CQD-1 on cells differentiation

**Fig. 5:**
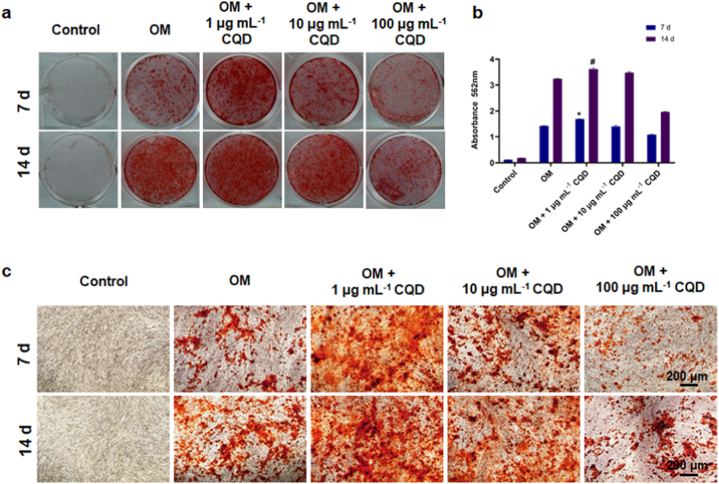
Effects of CQD-1 on cell differentiation. (a) ARS staining and (b) quantification of mineralized nodules on day 7 and day 14. hBMSCs stimulated with different concentrations of CQDs culture medium with OM. Control: hBMSCs cultured with regular medium without OM. *: P < 0.05 versus OM group on day 7, #: P < 0.05 versus OM group on day 14. 1 μg mL^−1^ CQD-1 with osteogenic medium significantly enhanced osteogenic activity in hBMSCs and had a significantly higher calcium deposition on day 7 and day 14, compared with control and osteogenic induction medium groups. (c) ARS staining on day 7 and day 14 under microscopy shows that the quantity of the mineral nodules generated by MSCs in the OM + 1 μg mL^−1^ CQDs group was larger than the OM group at both time points.

The differentiation potential is another key characteristic for stem cells. Next, we evaluated effects of CQD-1 on the osteogenesis ability of hBMSCs *in vitro*. ARS staining (Fig. 5a) and calcium deposit quantitative analysis (Fig. 5b) showed that 1 μg mL^−1^ CQD-1 with osteogenic medium significantly enhanced osteogenic activity in hBMSCs and had a significantly higher calcium deposition on day 7 and day 14, compared with control and osteogenic induction medium groups. Microscopic analysis using ARS staining demonstrated that the quantity of the mineral nodules generated by MSCs in the OM + 1 μg mL^−1^ CQDs group was larger than the OM group at both time points (Fig. 5c). Jin and co-workers investigated that CQDs from ascorbic acid could promote the ability of bone regeneration *in vitro* and also repair calvarial bone defects in mouse model [38]. Moreover, aspirin-based CQDs [37] and citric acid-based CQDs [39] upregulated expression of osteoblast gene markers, including ALP, RUNX2, OCN, and BSP, and the resulting enhanced matrix mineralization. In line with this study, our findings illustrated that CQDs from *S. oleracea* activated cellular function of hBMSCs for osteogenic differentiation for further application in bone regeneration at high concentrations and low toxicity.

### 3.6 Acute toxicity induced by CQD-1 *in vivo*

**Fig. 6:**
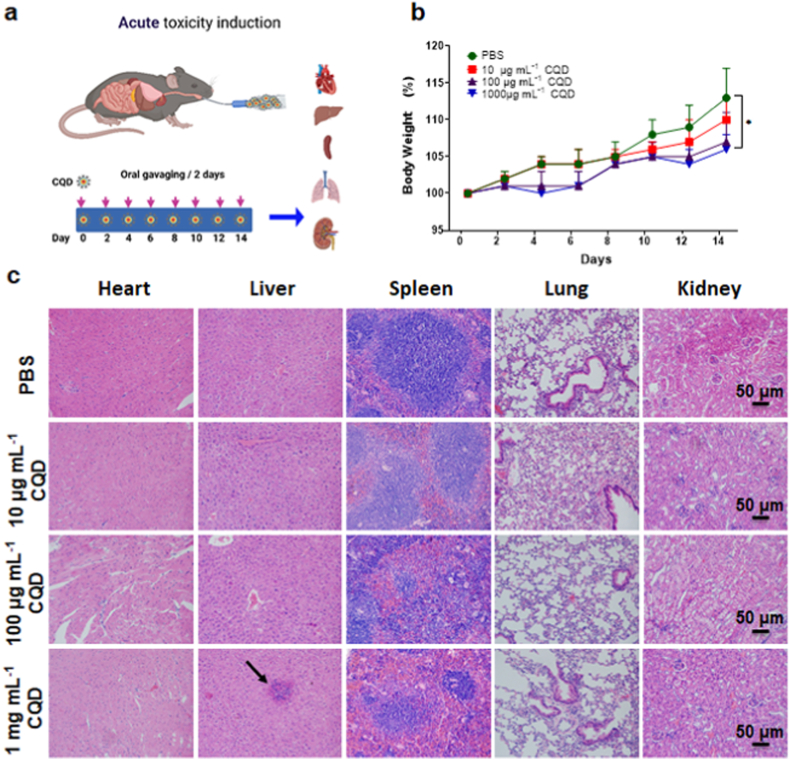
Acute toxicity induced by CQD-1 *in vivo*. (a) Schematic illustration of the toxicological evaluation. (b) Percentage of body weight change. *: P < 0.05 versus PBS group on day 14. After 2 weeks of oral gavage, all mice survived and no significant body weight growth was observed among the PBS group, 10 μg mL^−1^, and 100 μg mL^−1^, while treatment with 1 mg mL^−1^ CQD-1 significantly slowed the body weight growth in mice. (c) Histological assessments of H&E staining in the main organs of mice after treatment with PBS as control and different concentrations of CQD-1 (PBS, 10 μg mL^−1^ CQD, 100 μg mL^−1^ CQD, 1 mg mL^−1^ CQD). Arrow: lymphocytes aggregates.

To study the *in vivo* toxicity, we used the acute toxic model to evaluate the toxicity of CQD-1 in mice. Acute systemic toxicity testing provides the basis for hazard labeling and risk management of chemicals [47]. We evaluated the biosafety of CQDs *in vivo* as scheme (Fig. 6a). All substances are potentially toxic at sufficiently high concentrations [48,49]. Therefore, mice were treated with 3 different doses of CQD-1 (10 μg mL^−1^, 100 μg mL^−1^, and 1 mg mL^−1^) to investigate the *in vivo* toxicity. After 2 weeks of oral gavage, all mice survived and no significant body weight growth was observed among the PBS group, 10 μg mL^−1^, and 100 μg mL^−1^, while treatment with higher doses (1 mg mL^−1^) of CQDs significantly decreased the body weight growth in mice (Fig. 6b). To study the organ damage in the acute stages, histological analyses were conducted by H&E staining of the main organs. After treatment with various formulations for the four groups, as illustrated in Fig. 6c, no tissue necrosis was observed in the main organs (heart, liver, spleen, lung, and kidney) when the concentration was below 100 μg mL^−1^, which is similar to other reports [50]. Whereas above 1 mg mL^−1^, there were lymphocyte infiltrations found in the liver (Fig. 6c), which illustrated there was inflammation in livers after 14 days oral gavage of CQDs, while lots of dark spots with tens of micrometers in diameter appear in the livers and spleens of BALB/c mice after 2 weeks injection of graphene quantum dots [51]. It demonstrated that the CQDs from *S. oleracea* seemed to have no visual biological toxicity. Taken together, none of the tested organs showed significant histological lesions after 2 weeks of CQDs acute toxicity. While with the highest concentration of 1 mg mL^−1^ CQDs continuous oral gavage for 2 weeks, there was immune response that caused damage to livers. These CQDs show low toxicity *in vivo*, which suggests that CQDs produced during cooking *S. oleracea* are relatively safe and make them a candidate for further biomedical application.

## 4 Conclusion

Our work has an important role in evaluating food safety and promoting the biological application of food-derived CQDs. In summary, the study focused on the generation of microwave-assisted production of S. oleracea CQDs with different particle sizes and surface groups. CQD-1 from juicer with higher *QY* value is selected for toxicological analysis. The *in vitro* cell imaging results revealed that the CQDs can enter into the cytoplasmic of the hBMSCs, while cell viability *in vitro* and acute toxicity evaluation *in vivo* revealed virtually no-toxic effect up to 100 μg mL^−1^. Only high concentration of CQD (1 mg mL^−1^) induced cell apoptosis after 1 day. 1 μg mL^−1^ of CQDs enhanced the osteogenic ability of hBMSCs *in vitro* on day 7 and day 14. At the lower doses tested none of the major organs showed significant histological lesions after 2 weeks as a result of CQDs toxicity *in vivo*. In contrast, when using the highest concentration of 1 mg mL^−1^ an immune response, causing liver damage, was observed. These results indicate that food-derived CQDs have potential for safe biological applications at low concentrations. The toxicity investigation shows us a new approach to evaluating CQDs which can be used as potential therapeutic agents.

## Author contribution statement

Cuicui Fu: Conceived and designed the experiments; Performed the experiments; Analyzed and interpreted the data; Wrote the paper.

Xiaoyun Qin: Conceived and designed the experiments; Analyzed and interpreted the data; Contributed reagents, materials, analysis tools or data; Wrote the paper.

Jin Zhang; Yeqing Song; Jiaqi Yang: Performed the experiments.

Ting Zhang: Performed the experiments; Contributed reagents, materials, analysis tools or data.

Gang Wu: Contributed reagents, materials, analysis tools or data.

Dan Luo: Conceived and designed the experiments.

Nan Jiang; Floris J. Bikker: Conceived and designed the experiments; Contributed reagents, materials, analysis tools or data; Wrote the paper.

## Funding statement

Nan Jiang was supported by 10.13039/501100005090Beijing Nova Program [Z201100006820080].

Xiaoyun Qin was supported by 10.13039/501100001809National Natural Science Foundation of China [21904120], Henan Provincial Science and Technology Research Project [212102310858].

Dan Luo was supported by 10.13039/501100001809National Natural Science Foundation of China [51902344].

## Data availability statement

Data included in article/supp. material/referenced in article.

## Declaration of competing interest

The authors declare that they have no known competing financial interests or personal relationships that could have appeared to influence the work reported in this paper.
