# Supplementary material for: In vitro and in vivo toxicological evaluation of carbon quantum dots originating from Spinacia oleracea
Source: Heliyon. 2023 Feb 2;9(2):e13422. doi: 10.1016/j.heliyon.2023.e13422 (PMC9937992; doi:10.1016/j.heliyon.2023.e13422)
Supplement: Multimedia component 1 [file mmc1.docx]

**Supplementary Information**

***In vitro* and *in vivo* toxicological evaluation of carbon quantum dots originating from *Spinacia oleracea***

Cuicui Fu ^1^†, Xiaoyun Qin ^2^†, Jin Zhang ^2^, Ting Zhang ^3^, Yeqing Song ^4^, Jiaqi Yang ^5^, Gang Wu ^6,7^, Dan Luo ^8,9^*, Nan Jiang ^4^*, Floris J. Bikker ^1^*

1. Department of Oral Biochemistry, Academic Centre for Dentistry Amsterdam (ACTA), University of Amsterdam (UvA) and Vrije Universiteit Amsterdam (VU), Amsterdam 1081LA, the Netherlands
2. School of Material and Chemical Engineering, Zhengzhou University of Light Industry, Zhengzhou 450002, China
3. Laboratory of Biomimetic Nanomaterials, Department of Orthodontics, Peking University School and Hospital of Stomatology, National Engineering Laboratory for Digital and Material Technology of Stomatology, Beijing Key Laboratory of Digital Stomatology, Beijing 100081, China
4. Central Laboratory, Peking University School and Hospital of Stomatology, #22 Zhongguancun South Avenue, Haidian District, Beijing 100081, China
5. Shanxi Medical University School and Hospital of Stomatology& Shanxi Province Key Laboratory of Oral Diseases Prevention and New Materials, Shanxi 030605, China
6. Department of Oral and Maxillofacial Surgery/Pathology, Amsterdam UMC and Academic Center for Dentistry Amsterdam (ACTA), Amsterdam Movement Science, Vrije Universiteit Amsterdam, Amsterdam 1081LA, the Netherlands
7. Department of Oral Cell Biology, Academic Center for Dentistry Amsterdam (ACTA), University of Amsterdam and Vrije Universiteit Amsterdam, Amsterdam 1081LA, the Netherlands
8. CAS Center for Excellence in Nanoscience, Beijing Key Laboratory of Micro-nano Energy and Sensor, Beijing Institute of Nanoenergy and Nanosystems, Chinese Academy of Sciences, Beijing 101400, China
9. School of Nanoscience and Technology, University of Chinese Academy of Sciences, Beijing 100049, China

† These authors contributed equally.

***Corresponding authors.** E-mail addresses: [luodan@binn.cas.cn](mailto:luodan@binn.cas.cn) (Dan Luo), [nanjiang@bjmu.edu.cn](mailto:nanjiang@bjmu.edu.cn) (Nan Jiang), [f.bikker@acta.nl](mailto:f.bikker@acta.nl) (Floris J. Bikker).

**
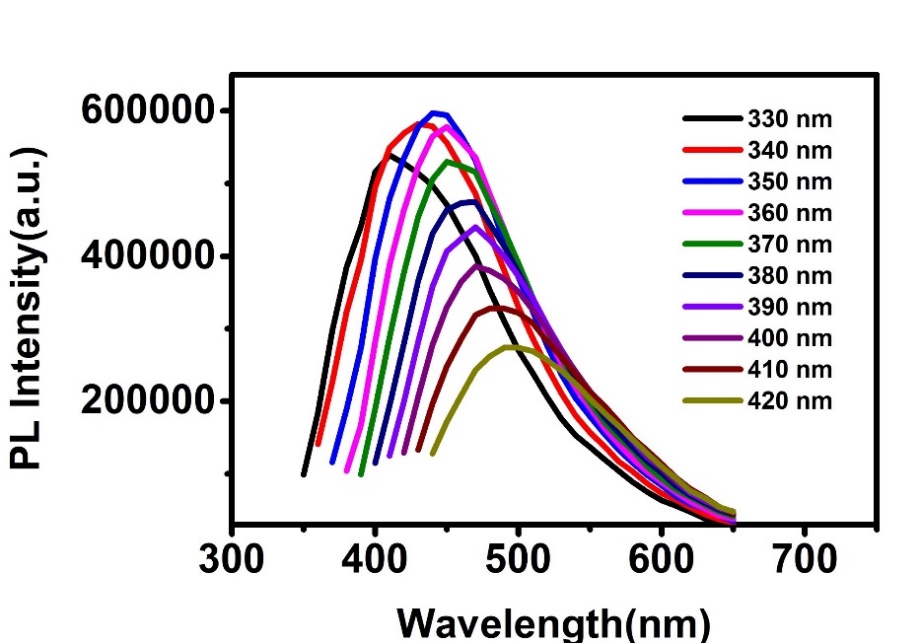
**

**Fig. S1** **PL emission spectra of CQD-1.** The excitation-dependent PL emission spectra of the aqueous dispersion of CQD-1 (10 μg mL^-1^, slit width 10☓10). The right column exhibits the excitation wavelengths for the same CQD sample.

**Table S1** **The atomic percentage of the two CQDs.**

|  | C | O | N | S |
| --- | --- | --- | --- | --- |
| CQD-1 | 78.35 at% | 19.78 at% | 1.72 at% | 0.15 at% |
| CQD-2 | 79.91 at% | 18.02 at% | 1.94 at% | 0.13 at% |

**Table S2** **XPS of CQDs.** C 1s, O 1s, N 1s, and S 2p peak assignments, binding energies, full width at half maximum, and emission area ratios.

|  | **C 1s** | **B.E(eV)** | **FWHM(eV)** | **Area%** |
| --- | --- | --- | --- | --- |
| CQD-1 | C=O | 288.78 | 1.19 | 8.3% |
|  | C-O | 286.18 | 1.9 | 31.4% |
|  | C-C | 284.77 | 1.26 | 60.3% |
| CQD-2 | C=O | 288.80 | 1.04 | 8.2% |
|  | C-O | 286.34 | 1.47 | 26.5% |
|  | C-C | 284.82 | 1.26 | 65.3% |
|  | **O 1s** | **B.E(eV)** | **FWHM(eV)** | **Area%** |
| CQD-1 | O=C-O | 534.13 | 1.16 | 6.9% |
|  | C-OH | 533.36 | 1.19 | 26.9% |
|  | O=C-O | 532.21 | 1.88 | 66.2% |
| CQD-2 | O=C-O | 533.81 | 1.19 | 11.8% |
|  | C-OH | 533.21 | 1.03 | 22.7% |
|  | O=C-O | 532.09 | 1.64 | 65.5% |
|  | **N 1s** | **B.E(eV)** | **FWHM(eV)** | **Area%** |
| CQD-1 | -NH_2_ | 400.47 | 0.5 | 6.4% |
|  | N-(C)_3_ | 399.87 | 0.66 | 32.8% |
|  | C-N-C | 399.31 | 0.74 | 55.4% |
|  | C=N-C | 398.69 | 0.5 | 5.4% |
| CQD-2 | N-(C)_3_ | 399.78 | 1.04 | 37.2% |
|  | C-N-C | 399.37 | 0.78 | 56.4% |
|  | C=N-C | 398.75 | 0.56 | 6.4% |
|  | **S 2p** | **B.E(eV)** | **FWHM(eV)** | **Area%** |
| CQD-2 | 2p_1/2_ | 168.78 | 1.32 | 64.7% |
|  | 2p_3/2_ | 167.85 | 0.81 | 35.3% |
| CQD-1 | sulfate | 169.71 | 0.57 | 13.7% |
|  | 2p_1/2_ | 168.78 | 0.88 | 30.6% |
|  | 2p_3/2_ | 167.76 | 0.89 | 55.7% |
